# Major research prize awarded to wheat researchers

**DOI:** 10.1111/pbi.12909

**Published:** 2018-03-25

**Authors:** Peter Shewry

**Affiliations:** ^1^ Rothamsted Research Harpenden Hertfordshire AL5 2 JQ UK

Most readers will be familiar with Professor Keith Edwards (University of Bristol), who was Founding Editor of the Plant Biotechnology Journal, steering the journal from 2002 to 2012. During his 10‐year tenure as Editor‐in‐Chief, Keith also led an internationally leading research programme on applied wheat genomics, and this aspect of his work has now been recognized by the award, jointly with Professor Graham Moore of the John Innes Centre (Norwich), of the Rank Prize for Nutrition. This award, valued at £80 000, recognizes their work which has enabled breeders to exploit variation in the wheat genome for crop improvement.

The Rank Prize for Nutrition is one of two prizes awarded by the Rank Prize Funds, the second being in the field of optoelectronics. These topics reflect the two major commercial interests, milling and baking and film production and distribution, of J. Arthur (Baron) Rank, who established the Funds shortly before his death in 1972. However, the milling and baking business has deeper roots, being built on the business established by his father Joseph Rank (1854–1943). He was a Yorkshire miller who revolutionized the UK wheat milling industry in the mid‐1880s, by introducing roller milling from Central Europe and by replacing wind and water power with steam power, allowing his mills to be moved close to the docks to facilitate the use of imported wheat. He was therefore largely responsible for enabling ordinary working people to consume white bread, which had previously been a luxury. It is therefore fitting that of the 31 the Rank Prizes awarded for Nutrition, five have been for research focused on or related to wheat.

Graham Moore's early work established the concept of ‘genetic synteny’, the similarity in the organization and sequences of the genomes of wheat and other cereal species. This breakthrough changed the way that we approach cereal genetics and genomics, and in particular, has allowed information from species with relatively simple genomes (such as rice) to be exploited to understand the highly complex wheat genome. Following this, he elucidated the mechanism of the ‘pairing locus’ which stabilizes the polyploid genomes of hexaploid bread wheat and tetraploid durum wheat, by preventing mispairing, and exchange between related sets of chromosomes during meiosis. This has facilitated the use of mutations in the locus to transfer genes for useful traits from related species. Finally, Graham has led the development of the UK public sector wheat prebreeding programme which brings together the major wheat research groups, including that of Keith Edwards, to deliver improved lines and tools for breeders in the UK and internationally.

Keith Edwards has led the development of new DNA marker systems for wheat breeding. To achieve this, he developed developing innovative approaches to reduce the complexity of the wheat genome by focusing on the small part of the genome which comprises expressed genes. He then exploited this information to develop high‐throughput methods to distinguish differences between and within the individual genomes of bread and durum wheats at the level of single DNA base changes. This has allowed the simultaneous determination of over 800 000 molecular markers in multiple wheat samples. Furthermore, he has developed a streamlined and cost‐effective ‘Breeders’ Chip’, widely used in commercial plant breeding, which determines variation in 35 000 DNA markers. Keith has also ensured that the tools and resources that he has established are available in accessible public repositories. Consequently, his CerealsDB website is the most widely used global source of data on wheat genetic diversity.

The prize therefore recognizes plant biotechnology in its widest sense, with high‐quality basic science underpinning applications with wide impacts on wheat improvement.

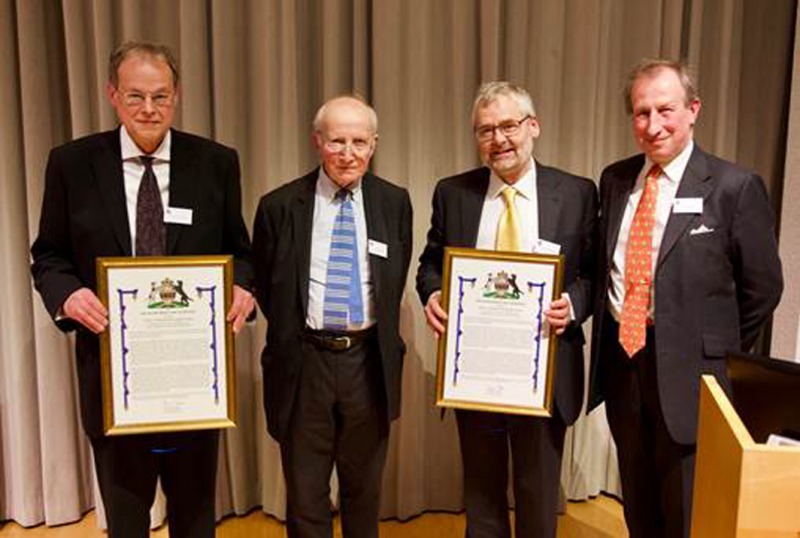

Left to right, Professor Graham Moore, The Guest of Honour, The Earl of Selborne, GBE, FRS, Professor Keith Edwards and the Chairman of the Rank Prize Funds, Mr. Stuart Cowen.

